# Emotional experience is increased and emotion recognition decreased in multiple sclerosis

**DOI:** 10.1038/s41598-021-01139-z

**Published:** 2021-11-08

**Authors:** Line Pfaff, Daniel Gounot, Jean-Baptiste Chanson, Jérôme de Seze, Frédéric Blanc

**Affiliations:** 1 University of Strasbourg and French National Centre for Scientific Research (CNRS), ICube Laboratory UMR 7357 and FMTS (Fédération de Médecine Translationnelle de Strasbourg), Team IMIS/Neurocrypto, Strasbourg, France; 2 CIC (Clinical Investigation Centre) INSERM 1434 and Neurology Department, University Hospitals of Strasbourg, Strasbourg, France; 3 University Hospital of Strasbourg, Strasbourg, Biopathology of Myelin, Neuroprotection and Therapeutic Strategies, INSERM U1119, Strasbourg, France; 4 Geriatrics Department, University Hospitals of Strasbourg, CMRR (Memory Resources and Research Centre), Geriatric Day Hospital, Strasbourg, France

**Keywords:** Multiple sclerosis, Cognitive neuroscience, Emotion

## Abstract

Emotional disorders in multiple sclerosis (MS) are frequently described as difficulties in recognizing facial expressions, rarely in the experience dimension. Moreover, interaction between emotional disorders and cognitive or psychological disorders remains little documented. The aim of this study is to explore emotions in MS in emotion recognition and emotional experience and compare these data with cognitive, psychological, and disease aspects. Twenty-five women with MS (MS group) and 27 healthy controls (control group) matched for age, sex, and education were assessed for emotion recognition (Florida Affect Battery) and emotional experience (International Affective Picture System Photographs). Participants were also assessed for cognitive and psychological aspects. Compared to the control group, the MS group had more difficulty in recognizing emotions, and their subjective evaluations when presented IAPS pictures were more scattered, globally increased. Emotional dimensions were each correlated with executive functions but neither correlated with alexithymia, depression, anxiety, or MS characteristics. In conclusion, MS patients present difficulties in identifying emotion and their emotional experience appears to be increased. These disorders are correlated with cognition but remain independent of psychological or disease aspects. Considering the implications that emotional disorders may have, it seems essential to take these aspects into account in clinical practice.

## Introduction

Emotions are permanent and essential for the human real-life experience; they are essential to our survival and, moreover, they form the basis of our social life^1^. A real “guide” helping us to navigate in a complex world^2^,“they inform us about the relevant events for us, our survival, our needs, goals and values and help us to make decisions to better manage our personal and social life”^2^.

In multiple sclerosis (MS), emotional disorders were reported from the very first observation of the disease. Charcot (1868) described patients with “an almost stupid indifference in reference to all things. It is not rare to see them give way to foolish laughter for no cause, and sometimes, on the contrary, melt into tears without reason. Nor is it rare, amid this state of mental depression, to find psychic disorders arise which assume one or other of the classic forms of mental alienation”^3^ (translated by George Sigerson, p. 194–195, 1977). Nevertheless, studies concerning these aspects are few and recent, most likely because they were long considered to be mainly a psychological reaction to the disease Thus, the social consequences of the disease, as well as the unexpected nature of its evolution, are a risk factor for depression in MS patients^4^. However, independently of physical disorders, MS patients with brain involvement have more emotional symptoms compared to MS patients with spinal cord involvement. Furthermore, numerous studies have found correlations between emotional factors and brain damage^5^ suggesting that the disease itself promotes the development of emotional disorders.

Furthermore, with more than a hundred listed definitions^1^, emotion is an heterogeneous concept, and studies making reference to it do not attribute the same disorders according to the chosen definition.

In 2003, Montreuil and Petropoulo^44^ suggested differentiating between mood disorders and emotional disorders, the former consisting of a long-lasting modification of mood and the latter consisting of brief affective reactions. On the basis of this distinction, in MS the authors classified depression, anxiety, and pathological euphoria as mood disorders and classified alexithymia, a syndrome of pathological laughing and crying, emotional lability, and emotional incontinence as emotional disorders.

In the neurosciences, the behavioural aspects of an emotion, namely emotion recognition and the experience it elicits are frequently studied. Basic emotions theories, such as the one proposed by Ekman, differentiate a limited number of emotions, characterized as innate, easy, categorical, and immediate^2^. These emotions range along a bi-dimensional axis of valence (positive/pleasant, negative/unpleasant, neutral) and arousal (calm to intense) [see the circumplex theories; Russell (1980)]^6^. These emotions range along a bi-dimensional axis of valence: is the emotion positive/pleasant, negative/unpleasant, neutral? And axis of arousal; that is the intensity of the emotion: is it weak/calm or strong/intense? [See the circumplex theories; Russell (1980)]^6^ For example, the vision of a beautiful landscape is generally judged as having a positive valence but low intensity, while the vision of a serious road accident is evaluated as having a negative valence and high intensity”. The emergence of the affective neurosciences has also raised questions about the cognitive component of an emotion, by integrating emotion as the essential datum of social cognition, and its neurobiological substrate.

In this context, several studies have reported that MS subjects presented a deficit in emotional facial expression recognition^7–10^, a deficit that was more marked for negative emotions of fear, anger, or sadness^7–10^, whether in static conditions or in dynamic conditions closer to everyday life^11^. On the other hand, although the majority of studies have focused on facial emotion recognition, some studies have shown that these deficits could also concern other perceptive domains. Thus, in 2013, Kraemer et al. showed that MS subjects presented a deficit of prosody emotion perception and in particular during recognition of anger^12^.

On the contrary, other studies showed that, in MS, the capacity to recognize an emotion was preserved^13^ or dependent on cognitive functioning or mood^14^. However, emotional difficulties in MS seems to persist irrespective of the level of cognitive deficits or mood modifications and so seem to be a specific and independent disorder coexisting with a more diffuse dysfunction.

Finally, in terms of the emotional experience triggered by emotional stimulation, only a few studies have focused on patients with MS. Di Bitonto et al.^13^, for example, showed that MS patients had a less intense emotional real-life experience for negative content stimulations, both for images and for sounds, whereas the emotional real-life experience for positive and neutral stimulations did not differ from controls.

Emotional disorders in MS remain little documented and merit further exploration. The purpose of this study was to explore emotion in MS in its two neuroscientific dimensions, namely recognition and experience, and to look for possible links with cognition, mood, and clinical aspects of the disease. We added alexithymia to this exploration, because it constitutes a specific emotional disorder in MS. Our hypothesis was that MS patients would present emotional disturbances for unpleasant emotions, with a decreased capacity to recognize facial expression and a blunted emotional experience, and that these data could be linked with the alexithymia.

## Participants and methods

### Participants

Twenty-five right-handed female relapsing–remitting MS (RRMS) patients (MS group) were recruited in the Department of Neurology, Strasbourg University Hospital, France. As there are well-documented behavioural and physiological sex differences in emotional experience, it has been strongly recommended that these be taken into account in functional brain imaging and behavioral studies on emotion^15^. Consequently, to limit factors that could be a source of functional and clinical heterogeneity, we decided to include only women in our study.

Patients were required to have an Expanded Disability Status Scale (EDSS)^16^ score of 5 or less and be receiving β-interferon treatment. The diagnosis of RRMS was made by a neurologist, according to the 2005 revision^17^ of the original McDonald’s criteria^18^. Patients were matched for age and education level with 27 right-handed healthy women (control group), recruited by the “Centre d’ Investigation Clinique” of Strasbourg University Hospital or through an appeal for volunteers (see Table 1 for more information). Inclusion criteria applicable to all participants were: no history of major psychiatric disease or non-neurological disorders (other than MS in the case of the MS group); and normal or corrected visual acuity. The local ethics committee: COMITE DE PROTECTION DES PERSONNES EST-IV (CPP “EST-IV”) had approved the study and each participant signed a written informed consent. All methods were performed in accordance with the relevant guidelines and regulation of these committee CPP “EST-IV”.

**Table 1:** Demographic and clinical characteristics of RRMS patients and control group.

Demographic characteristics	MS group (*n* = *25*) *Mean *(*SD*)	Control group (*n* = 27) *Mean *(*SD*)	Significance of between-group difference
Gender	Female	Female	ns*
Age (years)	42.8 (9.9)	41.52 (10.0)	ns*
Education (years)	13.72 (2.1)	14.3 (3.1)	ns*
Disease duration (years)	10.36 (8.9)	N/A	
EDSS score	1.64 (1.57)	N/A	

*MS* multiple sclerosis, *RRMS* relapsing–remitting MS, *EDSS* Expanded Disability Status Scale, *SD* standard deviation, *N/A* not applicable.

*No significant between-group difference (p > 0.05).

### Neuropsychological assessment

#### Cognitive assessment

All participants were assessed with a comprehensive neuropsychological battery. Visual episodic memory was assessed using the 10/36 Spatial Recall Test (SPART)^19^ and delayed recall (SPART-DR). The Digit Span subtest of the Wechsler Adult Intelligence Scale-Fourth Edition (WAIS-IV)^20^ was used to assess short-term auditory verbal memory (Digit Span Forward) and auditory verbal working memory (Digit Span Backwards and Sequencing). Executive functions were assessed using tests of verbal phonological and semantic fluency^21^, the WAIS-IV Similarities subtest, and a computerized test from the Test of Attentional Performance (TAP)^22^, for inhibition and flexibility. For the latter task, reaction time and number of errors were chosen as the relevant criteria. For attentional functions, we used the Symbol Digit Modalities Test (SDMT)^23^, a 90-s oral processing speed evaluation task, and the TAP Divided Attention subtest. Pre-morbid IQ was estimated with a French version of the National Adult Reading Test (fNART)^24^.Verbal episodic memory was assessed using a 16-word learning task, called “RL/RI 16”^25^, comprising three phases: encoding, storage (with immediate and delayed total recall and a recognition phase), and recovery (with immediate and delayed total free recall).

#### Emotion assessment

##### Emotion recognition assessment

Facial emotion recognition was assessed using the Florida Affect Battery (FAB)^26^. This battery includes four emotional subtests appraising five basic emotions (happiness, sadness, anger, fear, and neutral), and one non-emotional subtest, named the facial identity discrimination test. This control phase was to make sure that participants did not suffer from perceptive difficulties or prosopagnosia. The emotional subtests (Emotional FAB) were: Facial Affect Discrimination (subtest 2), Naming (subtest 3), Selection (subtest 4), and Matching (subtest 5). Each subtest contained 20 trials and each emotion appeared five times. Their order was defined by the test and was the same for all subjects. In subtests 3, 4, and 5, it was possible to determine what emotion a subject erred on, and the emotion she incorrectly identified it as. For more details, see Bowers et al.^26^.

##### Emotion experience assessment

Participants had to view 100 emotional pictures selected from the International Affective Picture System (IAPS) database^27^ differing in valence (positive, negative, neutral) and arousal (ranging from calm to excited). During each presentation, the subject had to score her experience for the valence (on a scale from 1 “very negative” to 9 “very positive”) and for the arousal sensation (on a scale from 1 “very calm” to 9 “very excited”). Pictures were presented in a same valence and arousal block design, alternating between emotional and neutral blocks, in the same order for all subjects.

#### Self-report questionnaires

All subjects were asked to complete a French self-administered depression-scale named EHD^28^ measuring two dimensions of depressive mood in MS: loss of emotional control and emotional blunting.

All subjects were also screened for anxiety using the Hamilton Scale^29^ and for alexithymia using the French version of the 20-item Toronto Alexithymia Scale (TAS-20)^30^, including three subscales: difficulty identifying feelings (DIF), difficulty describing feelings to others (DDF), and externally oriented thinking (EOT).

Fatigue was assessed using the French version of the Modified Fatigue Impact Scale (MFIS)^31^, commonly used in MS and validated for this population.

On all these scales, the higher the score the more severe the symptoms.

### Statistical analysis

Statistical analyses were performed using Excel and SPSS-21. Non parametric statistical tests were used due to non-homogeneity of variance and non-normality of the distribution. A Mann–Whitney U test was performed for between-group comparisons of quantitative data and a Chi^2^ or Fisher Exact test for qualitative data, also depending on application conditions. A Wilcoxon test for paired samples was used for intra-group comparisons. An alpha level < 0.05 was considered statistically significant for between-group comparisons.

Emotion recognition accuracy was calculated from the result of subtests 2–5 and the type of error from the result of subtests 3–5.

For emotional experience, we analysed the mean scores (comparison of raw data) and the dispersion from those scores (analyse of the variability translated in distance to an attempted norm). This dispersion compares the score between the two groups, with regard to the standard deviation to the norm, established based on the control group’s scoring, (i.e.: we converted each score to a z score on the base to the mean and standard deviation from controls group).

Finally, the average distance from the norm (absolute z score) for the MS group and the control group, was calculated image by image.

For inter-group comparison, we compared the mean distance for valence and for arousal with regard to positive, negative, and neutral IAPS pictures.

In order to understand how the MS group differed with regard to the control group, we also quantified the positive and negative bias (e.g., number of positive and negative z scores) for the valence and the arousal components in the MS group with a Wilcoxon test for paired samples.

For the MS group, Spearman’s rho was used to explore the correlation between emotion recognition and emotional experience for the dispersion values with the facial identity test, cognition, mood, and disease characteristics. For these univariate analyses, an alpha level < 0.01 was considered statistically significant. A multivariate analysis was subsequently conducted with variables presenting an alpha level < 0.05, in univariate analysis and using emotion recognition as dependent variable. A square root transformation was made on the character studied if the Gaussian distribution assumption of the data was not checked.

## Results

### Facial identity discrimination

At the facial identity discrimination subtest of the FAB, MS patient present 98.60% (SD = 2.3) of correct answers and HC present 99.63% (SD = 1.3) of correct answers. No inter-group difference was found (p = 0.052), and all subjects have at least 19 correct answers out of 20.

### Emotion recognition

Compared to controls, MS patients gave significantly fewer correct answers on the Emotional FAB (*p* = 0.017).

**Figure 1:**
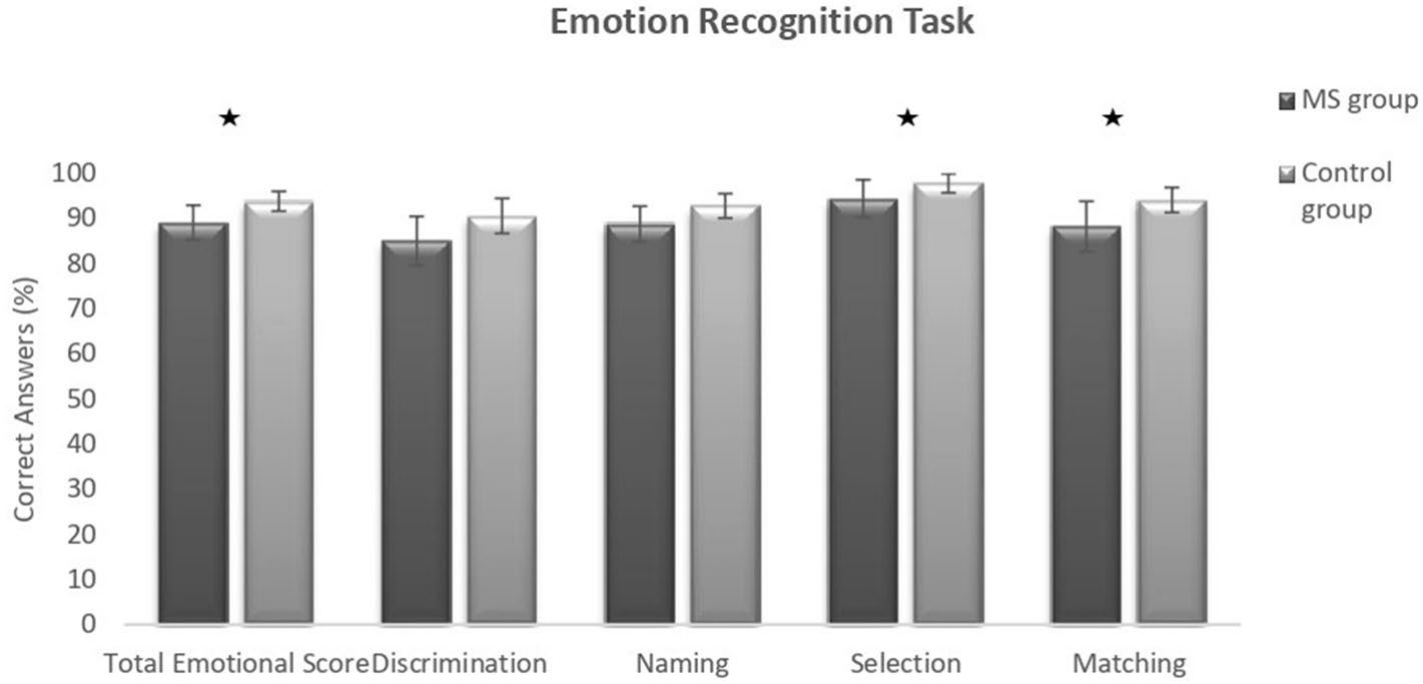
Emotion recognition task (FAB) results for the MS group and the control group. *Significant difference at *p* < 0.05.

MS patients had significantly more difficulties than control subjects on the Facial Affect Selection (*p* = 0.047) and the Facial Affect Matching subtests (*p* = 0.025). On the Facial Affect Discrimination and Facial Affect Naming subtests and on the Facial Identity Discrimination subtest no significant difference was found between the two groups (see Fig. 1).

An analysis of the types of errors made in the Facial Affect Discrimination subtest showed that patients made significantly more errors than control subjects in “anger-neutral” pairs (6 MS patients and none of the control subjects made at least one error (Χ^2^; *p* = 0.009)) and in “anger-sadness” pairs (8 MS patients and 2 control subjects made at least one error (X^2^; *p* = 0.036)).

On the Facial Affect Selection subtest, patients made significantly more errors with the neutral category, which they perceived as expressing an emotion (6 MS patients and 1 control subject made at least one error (X^2^; *p* = 0.018)).

MS patients also made significantly more errors by incorrectly interpreting fear as anger (4 MS patients and none of the control subjects made at least one error (X^2^; *p* = 0.047)).

On the Facial Affect Matching subtest, MS patients made significantly more mistakes with the neutral category, which they perceived as expressing an emotion (14 MS patients and 3 control subjects made at least one error (X^2^ *p* = 0.12)).

### Emotional experience

#### Between-group difference

##### Analysis of the mean score

The subjective evaluation of positive, negative, or neutral pictures did not differ between MS patients and control subjects for valence (unpleasant/pleasant) or for arousal when the raw values were compared between the two groups (see Table 2).

**Table 2:** Subjective evaluation of positive, negative, or neutral IAPS pictures: comparison of MS group and control group scores for valence and arousal.

	MS group Mean score (SD)	Control group Mean score (SD)	*p*-value
**Valence**			
Positive	7.45 (0.62)	7.24 (3.62)	0.22
Negative	2.76 (0.81)	3.03 (1.51)	0.10
Neutral	5.13 (0.44)	5.20 (2.60)	0.94
**Arousal**			
Positive	4.65 (1.90)	3.92 (1.80)	0.12
Negative	5.26 (1.70)	4.71 (1.49)	0.20
Neutral	1.84 (1.09)	1.51 (0.79)	0.30

**Figure 2:**
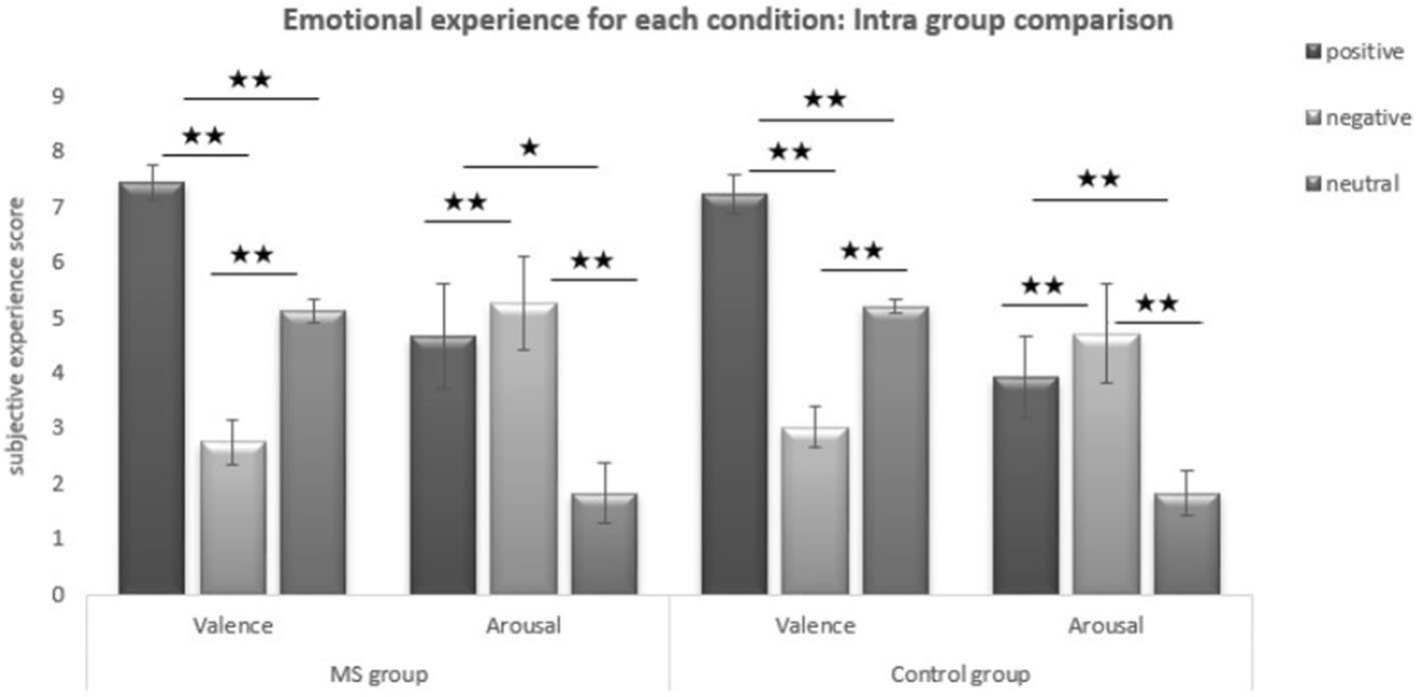
Intra-group comparisons of emotional experience in MS scoring for each condition. *Significant difference at *p* < 0.05; **Significant difference at *p* < 0.01.

Intra-group comparisons showed that both groups rated differently neutral in regard to positive and negative pictures and positive in regard to negative pictures, in both the valence and the arousal conditions (all *p* values < 0.05) (see Fig. 2).

##### Analysis of dispersion

In terms of valence, MS group scores were more scattered than those of the control group when presented with positive (*p* = 0.001), negative (*p* = 0.0036), and neutral (*p* = 0.016) IAPS pictures.

**Figure 3:**
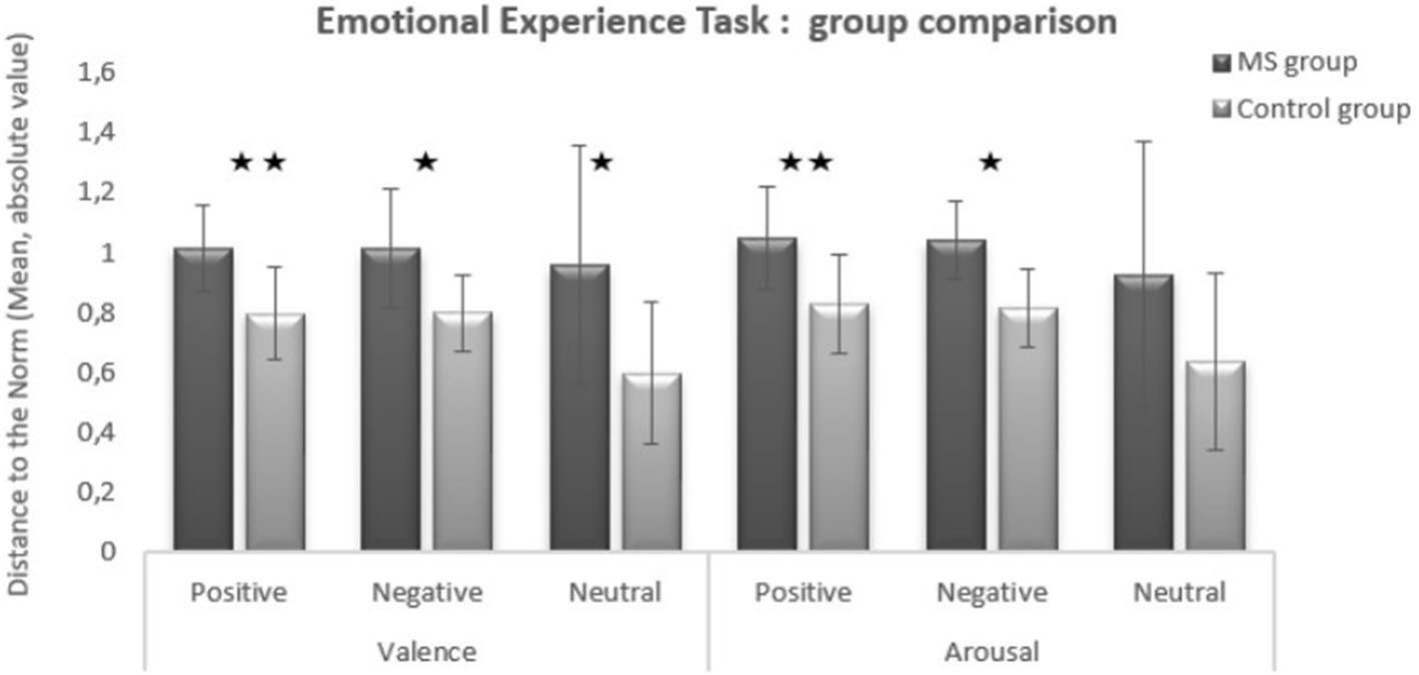
Dispersion of the subjective evaluation of emotional experience for positive, negative, and neutral IAPS pictures: comparison of MS group and control group scores for valence and arousal. *Significant difference at *p* < 0.05; **Significant difference at *p* < 0.01.

In terms of arousal, however, MS group scores were more scattered than those of the control group for positive (*p* = 0.024) and negative (*p* = 0.003) IAPS pictures, but not for neutral IAPS pictures (*p* = 0.097) (see Fig. 3).

### Response profile for MS group in comparison to controls’ norm for the dispersion

**Figure 4:**
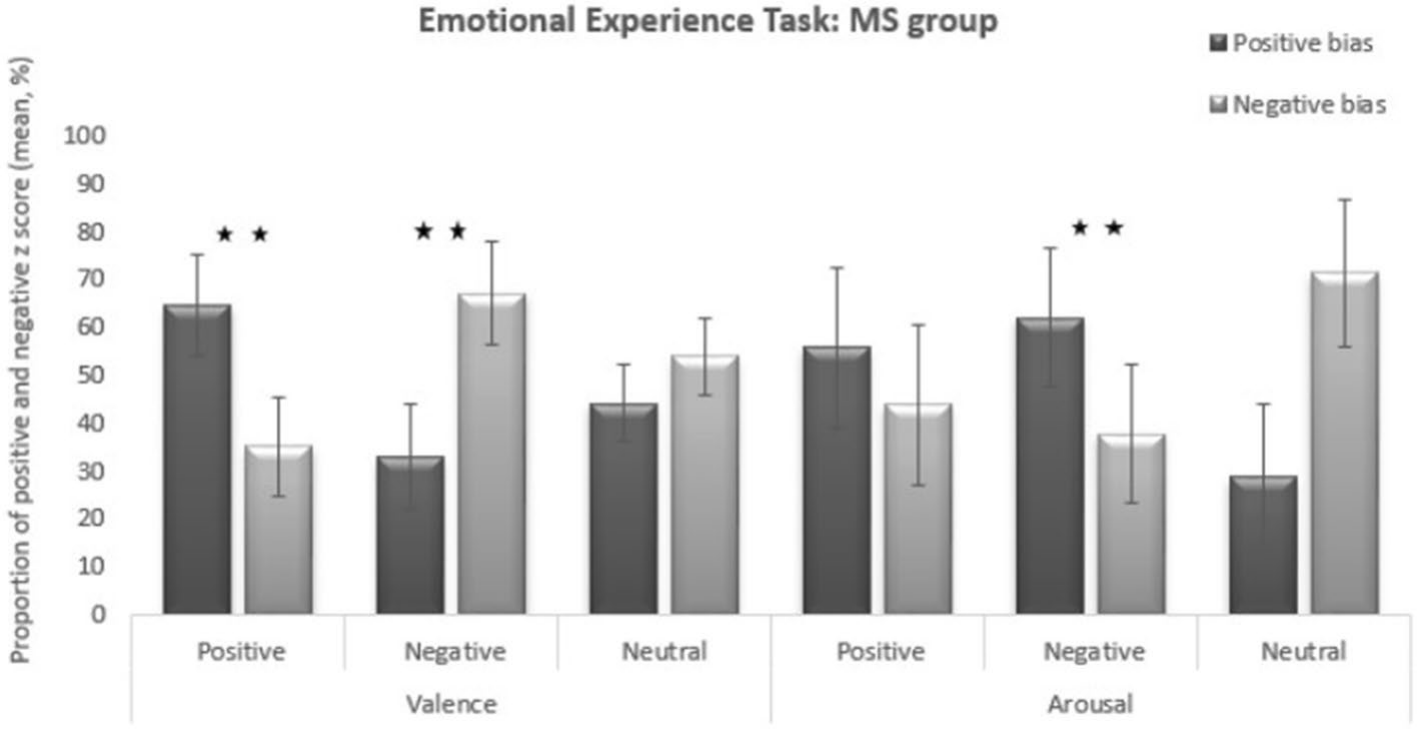
Subjective evaluation emotional experience for positive, negative, or neutral IAPS pictures: positive and negative bias in the MS group for valence and arousal (dispersion analysis). *Significant difference at *p* < 0.05; **Significant difference at *p* < 0.01.

An intra-group comparison was performed for the MS group to determine how their experience differed from that of controls (i.e., if patients’ z scores differed more often positively/higher or negatively/weaker when compared to the evaluation by controls) (see Fig. 4).

The results have to be interpreted taking account the 1 to 9 scale used during the debriefing. Thus, for the valence dimension, 1 corresponded to a very negative/unpleasant emotion, 9 to a very positive/pleasant dimension, and 5 to a relatively neutral emotion (not pleasant, not unpleasant). For the intensity dimension, whatever the valence dimension, 1 corresponded to a low intensity impact and 9 to a high intensity impact. Our results show that for the valence dimension, when presented with positive pictures, the MS group rated their experience significantly more often higher than controls on the 1 (unpleasant) to 9 (pleasant) scale (*p* = 0.002). When presented with negative pictures, the MS group rated their experience as significantly weaker on the 1 to 9 scale compared to controls (*p* = 0.004). When presented with neutral pictures, the MS group rated their experience as frequently higher and as frequently weaker when compared to controls (*p* = 0.399).

For the arousal dimension, negative pictures were rated more often higher in the MS group compared to controls (*p* = 0.004). Positive IAPS pictures were rated as frequently higher and as frequently weaker when compared to controls (*p* = 0.399). Neutral IAPS pictures were also rated as frequently higher and as frequently weaker when compared to controls (*p* = 0.106).

For the valence dimension, the MS group rated emotionally positive pictures as significantly more pleasant (positive bias) and emotionally negative pictures significantly more unpleasant (negative bias) compared to controls. For the intensity dimension, the MS group rated unpleasant pictures as more intensely unpleasant (positive bias) compared to controls.

### Cognitive assessment and self-report questionnaires

MS patients showed significantly slower processing speed, significantly lower scores on Digit Span Forward and Backwards, and significantly lower delayed recall on the 10/36 SPART compared to controls.

No significant group difference was found for any of the other assessed aspects of cognition (memory, executive functions, attention, Patients’ Premorbid IQ estimation are also equivalent to controls’ IQ estimation (see Table 3). MS patients had significantly higher scores than control subjects on the Depression, Anxiety, and Fatigue scales. MS patients also had higher alexithymia scores than control subjects on the DIF and EOT subscales but not on the DDF subscale (see Table 3).

**Table 3:** Cognitive assessment and self-report questionnaire: comparison of results for the MS group and the control group.

Neuropsychological assessement	Relevant criteria	MS group	Control group	*p*-value
		n = 25	n = 27	
		Mean Score (SD)	Mean Score (SD)	
**Memory**				
Verbal episodic memory	Encoding phase	15.40 (1.1)	15.74 (0.6)	> 0.05
16-word RL/RI task	Immediate free recall	30.76 (6.4)	32.37 (4.9)	> 0.05
	Total recall	46.8 (2.4)	47.26 (1.2)	> 0.05
	Recognition phase	16 (0)	15.93 (0.3)	> 0.05
	Delayed free recall	12.12 (2.9)	12.48 (1.6)	> 0.05
	Delayed total recall	15.80 (1)	15.89 (0.32)	> 0.05
**Visual episodic memory**				
10/36 Spatial Recall Test (SPART)	Immediate recall	15.96 (5.29)	19.44 (6)	0.033
	Delayed recall	5.32 (2.32)	5.81 (2.3)	> 0.05
Short-term memory	Digit Span Forward	5.72 (1.2)	6.3 (1.2)	0.012
Working memory	Digit Span Backwards	4.04 (1.37)	4.96 (1.48)	0.039
	Sequencing Digit-Span	5 (1)	5.44 (1.6)	> 0.05
**Executive function**				
Inhibition (Incompatibility Subtest, TAP)	Number of errors	2 (3.6)	0.85 (1.99)	> 0.05
Difference incompatibility/compatibility phase	Reaction time	98.84 (93.99)	77.04 (62.34)	> 0.05
Flexibility (TAP)	Number of errors	2.16 (2.42)	1.70 (1.99)	> 0.05
	Reaction time	1219 (1215. 814)	782.4 (186.619)	> 0.05
Verbal abstraction	WAIS-IV Similarity subtest	9.4 (3)	9.8 (2)	> 0.05
Verbal fluency (number of words in 2 min)	Phonologic fluency	22.8 (7.7)	25.4 (9.2)	> 0.05
	Semantic fluency	31.6 (7.4)	35.15 (9.5)	> 0.05
**Attention**				
SDMT for information processing speed	Correct answers	52.8 (12.1)	61.04 (8.2)	0.017
Divided attention (TAP)	Omissions	3.52 (3.08)	1.96 (1.17)	> 0.05
**Global cognitive efficiency**				
IQ pre-morbid estimation	fNART Total Score	110.2 (8)	111.4 (6.2)	> 0.05
**Self-report questionnaire**				
Depression	EHD Total Score (/44)	22.04 (5.98)	15.44 (4.23)	< 0.0001
	EHD Emotional blunting (/16)	6.44 (2.06)	4.96 (1.22)	0.004
	EHD Loss of emotional control (/28)	15.60 (5.37)	10.48 (3.81)	< 0.0001
Anxiety	Hamilton Scale Total Score (/56)	18.45 (12.29)	8.037 (7.11)	0.001
	Psychic anxiety	8.95 (5.65)	4.7 (4.32)	0.005
	Somatic anxiety	9 (7.15)	3.33 (3.29)	0.001
Fatigue	MFIS (0–100)	45.17 (24.15)	20.22 (7.34)	< 0.0001
Alexithymia	TAS-20 Total Score (0–100)	54.28 (11.64)	40.22 (10.97)	< 0.0001
	TAS-20 DIF (0–35)	20.2 (5.97)	12.74 (5.11)	< 0.0001
	TAS-20 DDF (0–25)	14.12 (4.56)	12 (4.51)	> 0.05
	TAS-20 EOT (0–40)	19.96 (4.49)	15.48 (4.09)	0.001

*16 word RL/RI task* 16 word free recall/cued recall task, *TAP* test of attentional performance, *SDMT* Symbol Digit Modalities Test, *fNART* French version of the National Adult Reading Test, *EHD* Depressive Mood Scale (*Echelle d’Humeur Dépressive*), *MFIS* Modified Fatigue Impact Scale, *TAS* Toronto Alexithymia Scale, *DIF* difficulty identifying feelings, *DDF* difficulty describing feelings, *EOT* externally oriented thinking.

### Correlations between emotion recognition (FAB), emotional experience (IAPS pictures) and other measures in MS patients

No correlations were found between emotional FAB and any of the emotional experience components (in terms of dispersion), suggesting an independence between these two functions (see Table 4).

**Table 4:** Correlational analysis of patients in the MS group.

	Emotion recognition	Emotional experience					
	*Emotional FAB*	*IAPS Valence score*			*IAPS Arousal score*		
	FAB 2,3,4,5	Positive	Negative	Neutral	Positive	Negative	Neutral
Disease duration	0.023	0.335	0.377	0.224	0.387	0.166	0.216
EDSS score	0.073	0.154	0.305	0.190	− 0.320	− 0.133	− 0.045
FAB 1	0.273	− 0.0.74	− 0.198	− 0.296	0.049	0.099	− 0.173
EHD (TS)	0.138	0.106	0.110	0.136	− 0.377	− 0.072	− 0.211
Hamilton Scale (TS)	0.095	0.145	0.210	0.128	− 0.097	0.168	− 0.139
MFIS (TS)	− 0.078	0.347	0.407	0.307	− 0.063	0.210	− 0.036
TAS20 (TS)	− 0.002	0.227	0.035	0.300	0.241	0.066	0.044
Inhibition (RT)	− 0.462	0.418	**0.569**	0.215	0.134	0.111	0.026
Flexibility (Errors)	− 0.424	0.486	0.471	0.161	0.188	0.217	0.032
Verbal abstraction (SN)	0.353	− **0.515**	− 0.270	− 0.075	− 0.186	− 0.149	− 0.106
SDMT (C.A)	0.434	− 0.313	− 0.435	− 0.338	− 0.076	− 0.126	− 0.283
Divided attention (O)	− **0.564**	0.364	**0.605**	0.270	− 0.003	0.309	0.221

*TS* total score, *RT* reaction time, *O* omission, *C.A* correct answers, *IAPS* International Affective Picture System, *FAB* Florida Affect Battery, *EDSS* Expanded Disability Status Scale, *SDMT* Symbol Digit Modalities Test, *EHD* Depressive Mood Scale (*Echelle d’Humeur Dépressive*), *MFIS* Modified Fatigue Impact Scale.

Bold values indicate significant correlation at *p* < 0.01.

### Multiple regression analysis of emotion recognition in the MS group

In the multivariate analysis we used emotion recognition (FAB 2,3,4,5) as dependent variable with variables presenting an alpha level < 0.05 in univariate analysis. This concerned inhibition, flexibility, SDMT, and divided attention.

We found that both inhibition (*p* = 0.024) and divided attention (*p* < 0.0001) were predictive variables for difficulty in identifying facial emotion in MS.

In the multivariate analysis for emotional experience we used positive and negative valence score as dependent variable with variables presenting an alpha level < 0.05 in univariate analysis for each of them.

For positive valence this concerned inhibition, flexibility, and similitudes and for negative valence this concerned MFIS (Fatigue Scale), inhibition, flexibility, SDMT, and divided attention.

For the positive valence we found that verbal abstraction (*p* = 0.001) was a predictive variable for the dispersion/distance between the MS group and the control group in the positive valence IAPS score.

For the negative valence we found that divided attention (*p* = 0.022) was a predictive variable for the dispersion/distance between the MS group and the control group in the negative valence IAPS score.

## Discussion

Our exploration of the two components of emotions revealed an original emotional profile in MS, with diminution of emotion recognition, as well as an exacerbation of the emotional experience coexisting with a high alexithymia score.

These apparently contradictory emotional disturbances were linked to preserved executive function, but were shown to be independent of mood disorders or disease aspects.

### People with MS have difficulty in identifying facial emotion

Compared to controls, MS patients gave significantly fewer correct answers for the global emotional FAB score. More precisely, of the four subtests, two differed from controls in the total score (selection and matching subtests), and difficulties were also observed for certain facial emotions, even in the test for which no inter-group difference was observed. This suggests that emotion recognition deficits remain subtle in MS, and that a unique or global evaluation of this function does not always allow existing difficulties to be highlighted.

In particular, for facial affect discrimination, MS patients had difficulties in differentiating anger from neutral and sad faces. In the “facial affect naming” subtest, MS patients misinterpreted fear as anger. In the “facial affect naming” and “facial affect matching” subtests, MS patients had difficulties with neutral faces, which were interpreted as being faces with emotional content.

Anger and, more generally, emotions with a negative valence seemed to be particularly difficult to identify in the MS group. These results are congruent with previous studies, which found a specific deficit in the detection of fear, anger^7,9^, and sadness^8^ in MS.

Studies with healthy subjects have shown that emotions with negative valence, which have more numerous labelling terms and are more complex to identify than positive valence emotions^32^ are underlain by vaster cerebral networks, and specific emotions (such as anger and fear) involve different brain regions^33^.

Given the diffuse distribution of plaques within the brain, MS is likely to alter general as well as more specific processing.

However, not only did our MS patients have difficulties in correctly identifying negative emotions, they also misinterpreted neutral faces, which they perceived as emotional faces. This error cannot be imputed to perceptive confusion in a global impairment of recognizing faces, because this aspect, which was controlled in the Facial Identity Discrimination subtest of the FAB, did not differ from controls.

Besides, topographically distinct findings argue for specific processing networks for emotional recognition and perception of faces. Thus, it seems likely that MS patients may have a specific impairment in analysing, identifying, and differentiating between emotional expressions, leading them to see emotion in neutral faces.

### People with MS also have a more scattered and increased emotional experience

Our subjects visualized standardized IAPS pictures, chosen for their ability to induce neutral, positive/pleasant, or negative/unpleasant affect in different levels of arousal from calm to strong.

Our two groups had to assess the experience induced by these stimulations by reporting valence and arousal sensations.

The results obtained suggest that people with MS have a similar global profile to that of controls, with a rating that varies according to the category of the type of stimuli affiliation (positive, negative, neutral). Despite this, their evaluation appeared to be more scattered than that of the control population. Indeed, the MS group appreciated differently the valence for the pleasant and unpleasant pictures.

In particular, pleasant pictures were more frequently experienced as being more pleasant, and unpleasant pictures as being more unpleasant, by MS subjects compared with controls.

This sensitization of the emotional experience of valence also affected the arousal induced by negative pictures, which were experienced as more intense by MS subjects than by controls.

As previously reported by Di Bitonto et al.^34^, the present results confirm that MS subjects have a perturbation of their emotional experience.

Furthermore, as in the facial emotion recognition task, neutral pictures were more often perceived by our patients as having an emotional valence, and were also considered as more pleasant or more unpleasant when compared to controls.

This emotional intensification for all the explored categories (positive, negative, and neutral) co-existing with a deficit in facial emotion recognition has also been observed in Huntington’s disease^35^, and was interpreted as an compensatory enhancement of one’s own feelings due to the diminished perception of emotion in the other. However, the neural lesion pattern of Huntington's disease, primarily localized in the basal ganglia, could also explain this specific emotional pattern^36^.

Indeed, de Arcos et al.^37^ observed that users of stimulating drugs (e.g. cocaine) also have a sensitization in terms of pleasant-unpleasant perception whereas users of depressive drugs (alcohol, heroin, heroine-cocaine) experience emotions with a reduced valence rating, towards a neutral valuation for both positive and negative simulation. These differences may reflect the activation of different mesolimbic-cortical motivational circuits, with the involvement of the dopaminergic system during the use of stimulating drugs and the GABAergic system for depressive drugs^36^.

Moreover, it is already well known that the basal ganglia process emotional information through the thalamocortical limbic circuit^38^. The involvement of basal ganglia in the modification of the emotional experience has been observed in Huntington’s disease^39^ as well as in Parkinson’s disease^40^.

In MS, basal ganglia atrophy is associated with fatigue^41^ and slower processing speed^42^. It would not be surprising if this structure also intervened in the modification of emotional experience. fMRI studies should help to better understand the underlying mechanism of this specific emotional pattern.

### Emotional disorders and other variables

Another aim of this study was to explore the relationship between the emotional profile and clinical variables in MS.

A recent study has suggested that emotional disorders in MS are dependent on cognitive dysfunction^10^, while other studies have indicated that these deficits persist irrespective of the level of cognitive deficit^14^.

In our study, the only correlations found with the emotional component concerned cognitive functions.

Thus, divided attention correlated with the emotional FAB score and both inhibition and divided attention correlated with the negative valence score. Abstraction correlated with pleasant experience in the MS group and was the most predictive variable to explain this difference when compared to the control group. Divided attention was the most predictive variable to explain the unpleasant experience in the MS group. However, none of this executive function performance differed from that of controls, thus excluding the possibility that emotional disorder could be secondary to cognitive deficits, such as a frontal syndrome.

The link between emotions and executive functions is still debated in the literature, yet fMRI studies have shown that these two entities involve common cerebral fronto-subcortical networks. It therefore seems possible that the aforementioned correlations are the reflection of close cerebral networks implicated as well as in several emotion components as in executive function, such as the dorso-medial prefrontal cortex, which is known to be involved in general emotional processing^32^ and to have a role in cognitive control^43,44^.

Our correlational studies show an independence between alexithymia and emotional disorders for the MS group. Moreover, the emotional experience profile of our MS group did not qualitatively correspond to the expected alexithymia profile, which is normally expressed as a moderate valence and arousal rating when patients are asked to assess the level of pleasantness of affect-laden slides^44^. These results are in line with previous studies that suggest that alexithymia is not a good predictor of the ability to recognize facial emotions in MS^45,46^ perhaps because they do not share quite the same neurobiological processes^47^. For Luminet et al., alexithymia is a stable personality construct, which is expressed as a continuum of low to high alexithimia in the healthy subject and through different pathologies^48^. Yet, it has been suggested that the deleterious effect of alexithymia on emotional performances would appear only at a high level of this disorder^49^. It could be interesting to consider differences between high-and-low alexithymia in MS emotional profile.

Consistent with findings from previous studies, negative mood (depression, anxiety) from our MS-patients was not associated with emotion recognition performances^34,50^ or modification in emotional experience^34^. However, other studies have shown that depression or anxiety were predictive of emotional scores^51,52^. These contradictory results could be explained by the degree of severity of the mood disorder. Indeed, as pointed out by Berneiser et al.^51^ a significant proportion of their patients fulfilled the criteria for at least mild depression, while a small proportion of patients showed clinical symptoms of depression in Kraemer et al. ^12^ or Pottgen et al. (2013)^50^. This hypothesis is also in line with recent studies of patients with anxiety or major depression without neurological comorbidity, which show that the degree of severity of mood disorder plays an important role in affecting emotional processing^53,54^. For our study, major anxiety or depression was an exclusion factor, which allows us to minimize the effect of these confounding variables. Thus, all of these data seem to indicate that the emotional changes observed in our cohort are manifested here independently of negative mood. However, further studies focusing solely on these aspects seem necessary, in particular by comparing populations of MS patients with various degrees of severity of mood disorders.

Finally, none of the disease characteristics (severity, duration, fatigue, which is a core symptom of MS) showed a correlation with emotional disorders. Moreover, it is important to underline that these deficits were observed in a cohort with a short disease duration (mean 10 years) and relatively low physical disability (mean EDSS 1.64), suggesting that emotional disorders appear in the early stages of the disease. Our results are in line with most studies as shown by a recent meta-analysis^55^. It has also been shown that emotional symptoms worsen with the severity of the disability^45^, which is probably a reflection of a more diffuse cerebral deterioration, and which is not incompatible with the fact that these difficulties can occur very early.

### Emotional profile of MS patients

This study highlights an atypical emotional profile in MS, with a reduction in emotional identification, an exacerbation of the emotional experience with a high alexithymia score. The coexistence of contrary emotional characteristics, some of which are reduced (identification and alexithymia) and others augmented (experience), could be partially responsible for diminished quality of life in MS^56^.

To our knowledge, our study is the first to identify the coexistence of these three elements. The lack of correlation between emotional disturbances and disease aspects (duration, EDSS score) suggests that these modifications can appear very early, from the first stages of the disease, and that they are lasting. Moreover, deficits were observed in an MS group with few motor or cognitive deficits, and at a relatively early stage of the disease. Recent studies have documented cases of subjects presenting psychiatric symptoms as inaugural symptoms of MS^57–59^. In a princeps fMRI study, Passamonti et al.^60^ showed that despite the similarity of the performances of MS patients and controls during a facial emotion recognition task, patients showed differences in cerebral activations, suggesting a cerebral process of a compensatory mechanism effective at the clinical/behavioural level but already indicating a deficit of emotional processing at the cerebral level.

Thus, it seems that emotional disorders in MS are not only reactional or secondary to the advance of the disease. It seems far more likely that they are a central symptom of this pathology, present from the first stages of the disease. Considering the impact of emotional disorders on the quality of life of patients^11^, but also on their social and professional skills^61^, these data emphasize the need to look for such dysfunctions in clinical practice.

In this respect, modification in emotional experience can perturb the quality of decision making^62^ and alter social functioning^61^. Kleeberg et al.^63^ already showed that MS subjects have a decreased decision-making capacity in the Iowa task, associated with less emotional reactivity skin conductance response. We can suppose that the scattered emotional experience observed in our population can cause the aforesaid difficulties.

### Limitations and perspectives

The present study has potential limitations. Firstly, we decided to include patients with some symptoms of depression that could have interacted with the emotional pattern. However, we showed that there was no correlation, but rather independence, between the emotional disorders and depression. We tried to understand the effect of the progression of the disease by including patients with a wide variety of disease duration. It would, however, be interesting to study the emotional profile of the same patients throughout the course of the disease, from the early stage clinically isolated syndrome, by means of a longitudinal study.

Finally, all the patients in our MS group were receiving β-interferon, and some preliminary clinical studies suggested that this treatment might favour the onset of emotional disorders^64^. However, a more recent long-term study supported the absence of any emotional worsening in MS patients treated with β-interferon^65^.

## Conclusion

In conclusion, patients with MS have difficulties in identifying emotions in other people and their emotional experience appears more scattered, with a global exacerbation of their emotional response These disorders are correlated with some cognitive tasks related to executive function and they most likely share common cerebral network and they most likely share a common cerebral network, while they coexist independently with alexithymia, depression, and anxiety. Furthermore, neither the severity of the physical disability nor the duration of the disease seems to interact with these difficulties.

Considering the implications that emotional disorders may have for MS patients as well as their familial, social and professional entourage, it seems essential to take these aspects into account in clinical practice.

The next step will be the analysis of structural and functional brain MRI to better understand the neural basis of such emotional modifications in MS.
